# Developing a Temperature-Inducible Transcriptional Rheostat in Neurospora crassa

**DOI:** 10.1128/mbio.03291-22

**Published:** 2023-02-06

**Authors:** Cyndi Tabilo-Agurto, Verónica Del Rio-Pinilla, Valeria Eltit-Villarroel, Alejandra Goity, Felipe Muñoz-Guzmán, Luis F. Larrondo

**Affiliations:** a Departamento de Genética Molecular y Microbiología, Facultad de Ciencias Biológicas, Pontificia Universidad Católica de Chile, Santiago, Chile; b Millennium Science Initiative-Millennium Institute for Integrative Biology (iBIO), Santiago, Chile; University of Melbourne

**Keywords:** *hsp* promoters, heat shock, synthetic promoter, inducible promoter, *Neurospora crassa*, HSP, *Neurospora*, synthetic biology, transcription

## Abstract

Heat shock protein (HSP)-encoding genes (*hsp*), part of the highly conserved heat shock response (HSR), are known to be induced by thermal stress in several organisms. In Neurospora crassa, three *hsp* genes, *hsp30*, *hsp70*, and *hsp80*, have been characterized; however, the role of defined *cis* elements in their responses to discrete changes in temperature remains largely unexplored. To fill this gap, while also aiming to obtain a reliable fungal heat shock-inducible system, we analyzed different sections of each *hsp* promoter by assessing the expression of real-time transcriptional reporters. Whereas all three promoters and their resected versions were acutely induced by high temperatures, only *hsp30* displayed a broad range of expression and high tunability, amply exceeding other inducible promoter systems existing in Neurospora, such as quinic acid- or light-inducible ones. As proof of concept, we employed one of these promoters to control the expression of *clr-2*, which encodes the master regulator of Neurospora cellulolytic capabilities. The resulting strain fails to grow on cellulose at 25°C, whereas it grows robustly if heat shock pulses are delivered daily. Additionally, we designed two *hsp30* synthetic promoters and characterized them, as well as the native promoters, using a gradient of high temperatures, yielding a wide range of responses to thermal stimuli. Thus, Neurospora *hsp30*-based promoters represent a new set of modular elements that can be used as transcriptional rheostats to adjust the expression of a gene of interest or for the implementation of regulated circuitries for synthetic biology and biotechnological strategies.

## OBSERVATION

The filamentous fungus Neurospora crassa has been used as a model organism for the molecular dissection of diverse complex biological processes, such as cellulose degradation (1, 2), gene silencing (3–5), circadian rhythms (6–9), and photobiology (10). The broad set of available molecular tools in this organism is also extensive, including a knockout collection (11), selectable markers (12–15), CRISPR/Cas9 technologies (16), and inducible/constitutive promoters (10, 17, 18).

Different promoters induced by chemical signals have been developed in N. crassa, such as ones that can respond to glucose (19), quinic acid (QA) (20, 21), nitrogen (22–24), and copper (24, 25). Nevertheless, the utilization of physical cues as inducing signals has mainly focused on the use of promoters responding to light (18, 26, 27), whereas other signals, like temperature, have seldom been employed as modulators of transcriptional units in this organism.

The heat shock response (HSR) is an evolutionarily conserved protective mechanism triggered by high temperatures, which can also be induced by other stresses (28). Inside cells, the HSR leads to, among other cellular changes, an intense and rapid synthesis of proteins called heat shock proteins (HSPs) that act as chaperones. These proteins are well conserved in terms of features and functions, and some of them have been also shown to play roles in normal development (28, 29). In eukaryotes, the *hsp* promoters have revealed the presence of consensus heat-responsive sequences known as heat shock elements (*hse*; 5′-NTTCNNGAANNTTCN-3′). Heat shock factors (HSFs) are the proteins in charge of recognizing *hse* boxes, exhibiting similar DNA binding domains across eukaryotes (30).

The *hsp* promoters and their expression profiles have been well studied in diverse model organisms, including plants (31, 32), mammals (33, 34), and fungi (35–38). In several cases, *hsp* promoters have been successfully utilized for spatial and temporal control of gene expression (39–41), to promote heat stress tolerance in distinct organisms (42–45), in heterologous protein and chemical production (46, 47), or even for synthetic-circuit-based biosensors (48).

In N. crassa, three genes encoding the major HSP from each family have been described: *hsp30* (NCU09364) (49), *hsp70* (NCU09602) (50), and *hsp80* (NCU04142) (51). It has been reported that these three *hsp* genes are expressed in response to high temperatures (50, 52, 53) and that they bear putative *hse* regulatory elements in their promoter regions (49–51, 54). Despite this, further characterization of the *hsp* promoters in Neurospora has not been systematically conducted, nor have minimal aspects like dynamic ranges of expression and their tunability by discrete temperature changes been studied. Such analyses are not only relevant to better understand how Neurospora responds to thermal stimuli but can also yield valuable information as to which *hsp* promoter(s) can be adopted as viable and versatile inducible systems.

In this work, we sought to characterize the transcriptional responses of the *hsp30*, *hsp70*, and *hsp80* promoters by utilizing a destabilized codon-optimized luciferase (Luc), a well-known reporter for transcriptional dynamics in Neurospora crassa (55, 56). Indeed, the addition of a degron (PEST sequence) to firefly luciferase turns this real-time reporter into an excellent system to dissect promoters of interest, including their range of inducibility upon cognate stimuli. Thus, we assessed the regulation conferred by the full and resected versions of each promoter upon exposure to different temperatures. Because of their highly tunable regulation and low basal level of expression, we selected *hsp30*-derived ones to further delve into their expression dynamics by exposing them to a gradient of high temperatures and a variety of treatment times. The end result is an accurate profile of responses to diverse temperature stimuli. In addition, and as a proof of concept of their applicability, we utilized a resected *hsp30* promoter to control the expression of *clr-2*, which encodes the master transcription factor involved in cellulose degradation, resulting in heat shock-conditional growth. Finally, we designed two synthetic promoters based on multiple *hsp30* (*SP30*) putative heat response elements to generate modular versions of these sequences in order to avoid repeated induced point (RIP) mutations and also to facilitate future synthetic biology strategies.

*In toto*, the results provide new and detailed data on *hsp* responses to temperature in Neurospora, while also establishing *hsp30*-derived systems as versatile transcriptional rheostats for graded gene expression, expanding the existing repertoire of inducible promoters in filamentous fungi.

### Results: functional analysis of *hsp* promoters.

**FIG 1:**
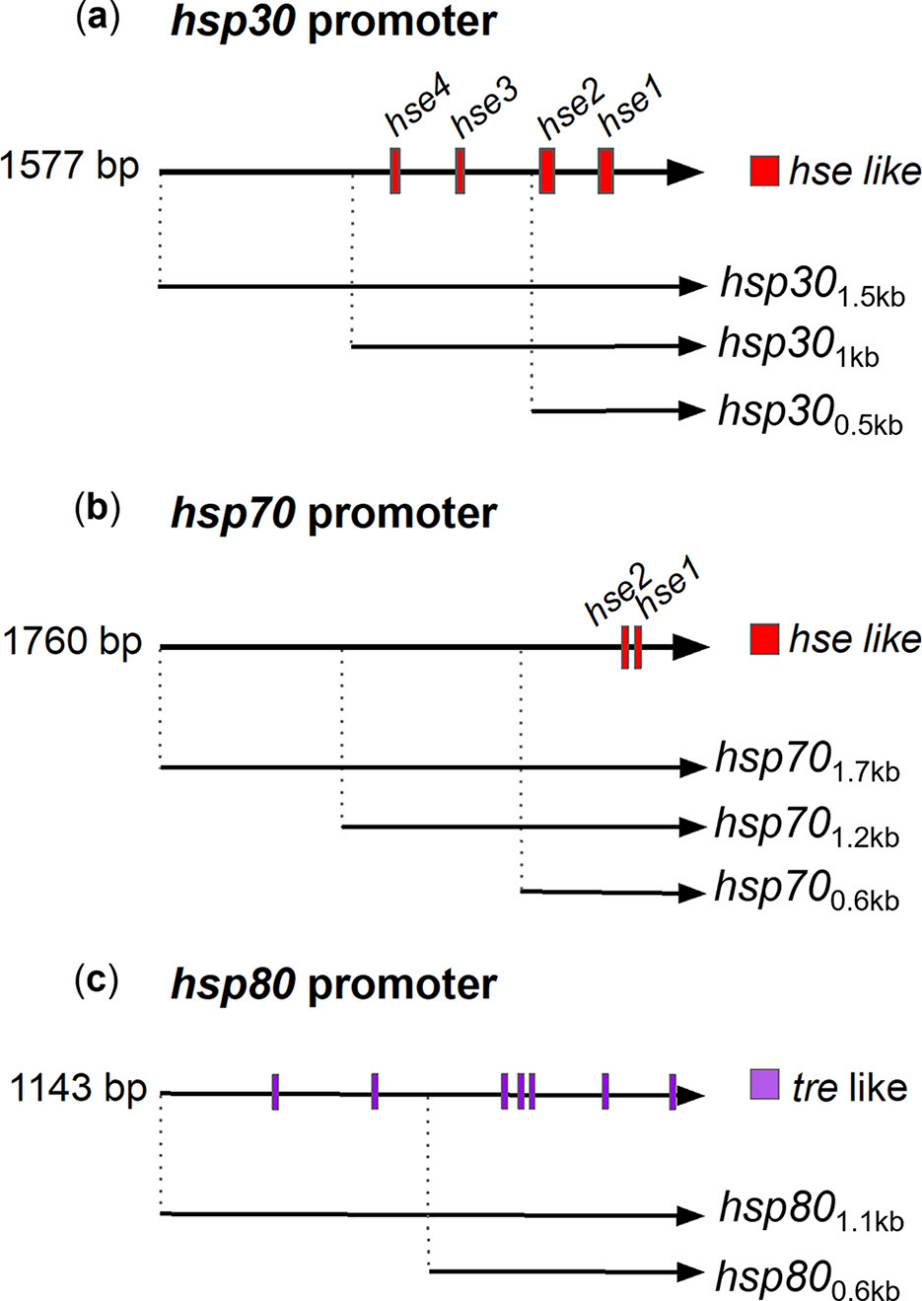
Putative transcriptional heat shock regulatory elements present in the *hsp* promoters. (a to c) Schemes of *hsp30*, *hsp70*, and *hsp80* promoters with the putative transcriptional regulatory elements indicated (49–51, 54). The *hsp30* and *hsp70* promoters contain putative heat shock elements (*hse*, red boxes), while the *hsp80* promoter bears putative temperature-responsive elements (*tre*, purple boxes). We analyzed the indicated sections, upstream from the ORF (arrowhead). The dimensions of the boxes and lines represent the sizes of the transcriptional regulatory elements and the promoter regions, respectively.

In previous reports, the sequences of the *hsp30*, *hsp70*, and *hsp80* promoters have been succinctly described (49–51). For *hsp30* and *hsp70*, four and two putative *hse* have been proposed (Fig. 1a and b), albeit none of them is a perfect match with the described consensus sequence (Table S1 at https://doi.org/10.6084/m9.figshare.21774671 and Table S2 at https://doi.org/10.6084/m9.figshare.21774632). For *hsp80*, previous studies did not report sequences resembling the consensus *hse* but, instead, described temperature response elements (*tre*) (Fig. 1c), which the authors proposed might allow the expression of this gene upon heat shock (51). However, the functionality of the regions containing these putative *cis* elements has not been experimentally confirmed in any of these promoters.

To advance such functional analyses, we generated an array of reporter strains spanning different promoter dissections of the above-mentioned *hsp* genes to control the expression of a destabilized firefly luciferase. The dissected regions comprise different lengths of upstream sequence (relative to the open reading frame [ORF]), which were selected depending on the presence of the putative heat-responsive elements (Fig. 1). Thus, we generated 8 dissections: three from full-length promoters for each *hsp* (*hsp30*_1.5 kb_, *hsp70*_1.7 kb_, and *hsp80*_1.1 kb_), based on the previously described sequences (49–51, 54), plus two resected versions for *hsp30* (*hsp30*_1 kb_ and *hsp30*_0.5 kb_), two for *hsp70* (*hsp70*_1.2 kb_ and *hsp70*_0.6 kb_), and one for *hsp80* (*hsp80*_0.6 kb_). The full-length and the 1-kb section of *hsp30* share the same four putative *hse*, while the smallest region has only two of those boxes. The *hsp70* promoter region contains the two previously proposed *hse* (Fig. 1b), whereas the *hsp80*_0.6 kb_ section keeps five of the seven potential *tre*-like elements (Fig. 1c).

**FIG 2:**
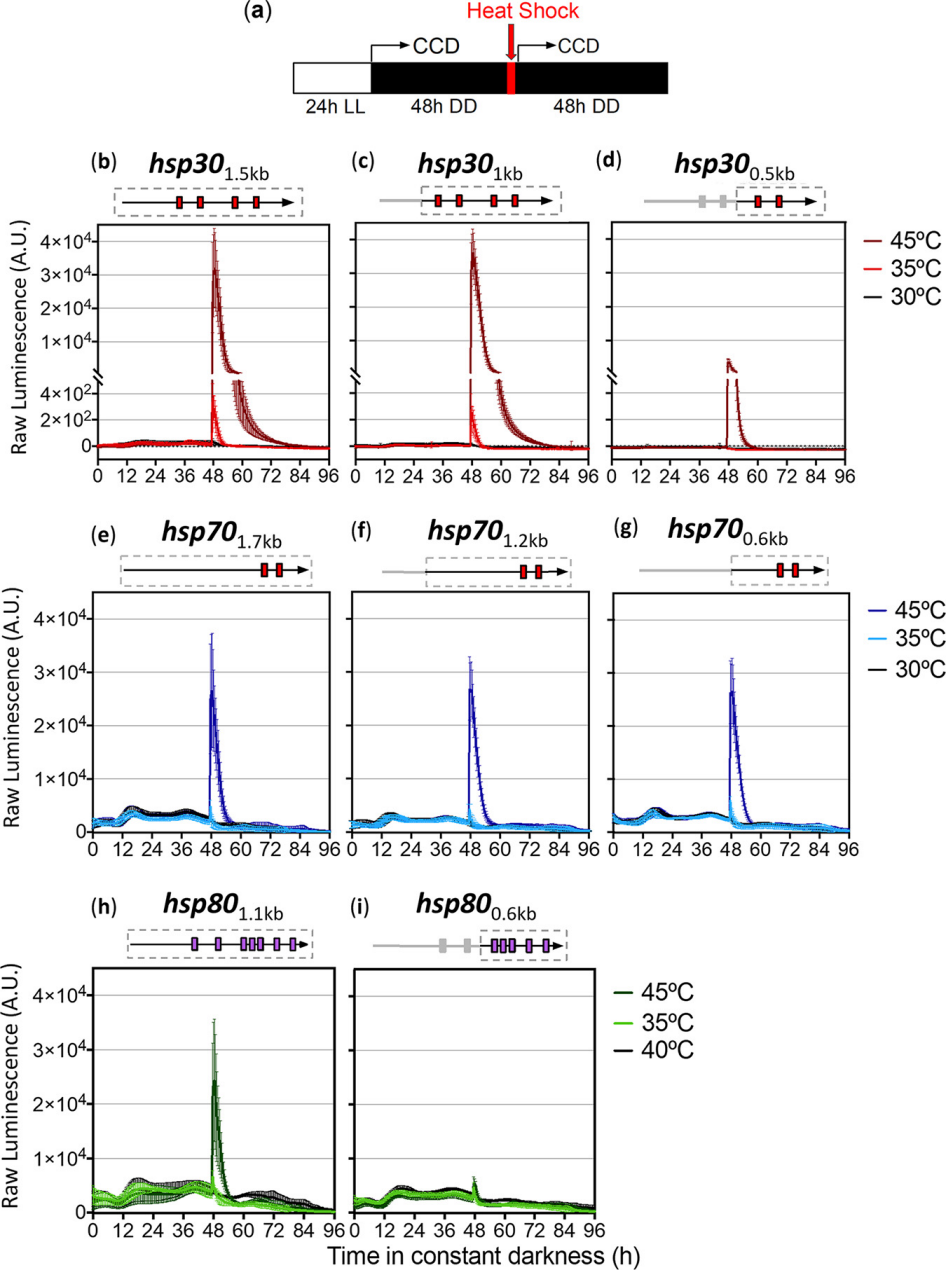
Luciferase activity profiles conferred by *hsp* promoters and resected sections upon heat shock treatment. (a) Description of the experimental setup. The bent arrows represent the start of the bioluminescence measurements using the CCD camera. The strains grew for 24 h at 25°C under constant light conditions (LL), and then we measured the luminescence at 25°C in constant darkness (DD). The heat shock treatments (30°C, 35°C, and 45°C) were delivered for 1 h using an incubator, after which luminescence was monitored for additional 48 h. (b to i) Luminescence levels are shown in arbitrary units (A.U.). Boxes in gray dotted lines above each chart represent the area of the promoter region being analyzed, whereas the red and purple boxes represent the putative *hse* and *tre*, respectively. Each curve corresponds to the average values from four to six biological clones with three independent wells each ± standard deviations (SD) and represents the behavior in two independent experiments.

As an exploratory analysis, we grew the reporter strains under constant light conditions (LL) for 24 h and then, using a charge-coupled-device (CCD) camera, recorded the luciferase activity of each promoter in darkness (DD) at 25°C (Fig. 2). After 48 h, we exposed them to 1 h of heat shock at three different temperatures: two high ones (35°C and 45°C) and one closer to Neurospora laboratory growth conditions (30°C). We observed that the three full-length promoters had similar fast and strong inductions at 45°C, while they displayed only reduced responses at 35°C and no changes were seen at 30°C (Fig. 2). The transcriptional profile of the resected *hsp30*_1 kb_ version was similar to that of *hsp30*_1.5 kb_ at all temperatures, whereas *hsp30*_0.5 kb_ showed reduced expression at 45°C, with less than half the levels of the full-length promoter, and no obvious induction at 35°C. The shorter versions of the *hsp70* constructs showed, at all tested temperatures, transcriptional profiles similar to that of the full version. The *hsp80*_0.6 kb_ region displayed equally diminished responses at both high temperatures, being about 9 times weaker than the response observed for *hsp80*_1.1 kb_ at 45°C (Fig. 2).

**FIG 3:**
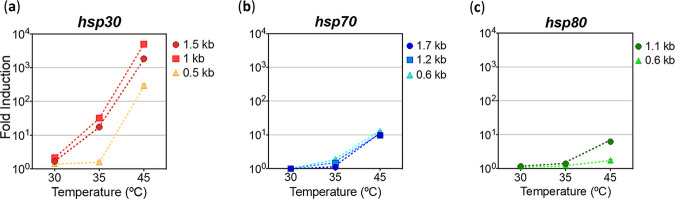
Fold induction achieved by the *hsp* promoter regions after heat shock treatments. (a to c) The fold induction (fold change) was calculated with the maximum luciferase expression, based on the average of the three highest consecutive values with respect to the background values before heat shock treatment of each promoter region. The data were obtained from the experiments whose results are shown in Fig. 2b to i. Average fold inductions are shown.

Comparing the overall expression profiles between the three *hsp* promoters, we observed that *hsp70* and *hsp80* exhibited higher basal activities at 25°C (Fig. S1 in the supplemental material). In contrast, the *hsp30* promoters had basal luminescence levels that were 10 and 15 times lower than those of the other two *hsp*, respectively, and were strongly induced after exposure to high temperatures. Indeed, the *hsp30* basal levels of expression were comparable to what is obtained when examining a lowly expressed gene like the clock gene *frequency* in its trough values (55, 56). This causes the *hsp70*- and *hsp80*-based promoters to have limited induction profiles after heat shock, measured as the fold induction between basal and peak levels of luciferase activity, while on the contrary, the *hsp30*-based promoters (full and resected constructs) displayed high fold induction ratios (Fig. 3). Thus, the *hsp30*_1.5 kb_ and *hsp30*_1 kb_ promoters exhibited ~10-fold and over 1,000-fold induction after being treated at 35°C and 45°C, respectively, whereas although the *hsp30*_0.5 kb_ promoter did not display a clear response at 35°C, it yielded an activation of over 100-fold when stimulated at 45°C.

10.1128/mbio.03291-22.1 FIG S1 Basal activities of *hsp* reporters. Basal (background) luminescence levels of each *hsp* promoter and their resected sections. Each bar indicates the average values from two or three biological clones with four independent wells each ± standard deviations (SD) and represents the behavior of two independent experiments. Values were obtained prior to delivering the heat shocks, in a 96-well-plate format (values are from the data sets from the experiments whose results are shown in Fig. 2). Download FIG S1, PDF file, 0.1 MB. Copyright © 2023 Tabilo-Agurto et al. 2023 Tabilo-Agurto et al. https://creativecommons.org/licenses/by/4.0/ This content is distributed under the terms of the Creative Commons Attribution 4.0 International license.

We then sought to compare the response of *hsp30* to those of other well-known inducible systems normally utilized in N. crassa, such as the *qa-2* promoter, which reacts to increasing concentrations of quinic acid (QA) (21), and the *vvd* promoter, which is activated by light (18). In our hands, *hsp30* gave inductions 10- to 1,000-times higher than the *qa-2* and *vvd* reporters, respectively (Fig. S2). Importantly, we could observe that *hsp30*_1.5 kb_ displayed lower background levels and maximum responses compared to the other promoters (Table S3 at https://doi.org/10.6084/m9.figshare.21774662). Additionally, regarding the time spanned to reach the highest response, the post- versus prestimulus levels, or the rate of signal decay, *hsp30*_1.5 kb_ showed several properties similar to those of the *vvd* promoter. *hsp30*_1.5 kb_ regained basal levels after stimuli at times ~2 to 3 h longer than those exhibited by the *vvd* promoter after a discrete light pulse. For the *qa-2* reporter, these aspects could not be evaluated, as QA remains in the medium after addition and, therefore, the response does not decrease after the stimulus is initiated. Importantly, the reporters gave different levels of induction in assays conducted in PCR tubes than in larger-volume tubes, and yet, in all cases, *hsp* exceeded the *qa-2-* and *vvd*-based systems. Considering all these characteristics, as well as the inducibility and tunability of the response of the *hsp30*-derived promoters, we further evaluated their behavior and tested their functionality.

10.1128/mbio.03291-22.2 FIG S2 Comparison of fold changes achieved by *hsp30* and other inducible N. crassa promoters. Fold inductions achieved by the *hsp30*, *vvd*, and QA (*qa-2*) promoters after providing the respective cognate stimuli. Fold induction was calculated with the maximum luciferase expression with respect to the average of the background values before inducing each promoter. Download FIG S2, PDF file, 0.1 MB. Copyright © 2023 Tabilo-Agurto et al. 2023 Tabilo-Agurto et al. https://creativecommons.org/licenses/by/4.0/ This content is distributed under the terms of the Creative Commons Attribution 4.0 International license.

### Detailed charting of the *hsp30* promoter responses to different heat shock stimuli.

We then performed a detailed functional characterization of the transcriptional responses of the different *hsp30* promoters upon discrete temperature changes within a 35 to 45°C range during different exposure times. To analyze this, we adopted a simple and yet practical strategy that allowed us to expose the Neurospora reporter strains to a heat shock gradient using a 96-well plate format (Fig. S3). We used arrays made with PCR tubes and a gradient thermal cycler to expose the cultures simultaneously to six different temperatures, 34°C, 36°C, 38°C, 40°C, 42°C, and 44°C, and to different treatment times, 60, 30, 15, 5, and 1 min. This approach allowed us to obtain faster and more accurate results than with the previous strategy (Fig. 2), providing precise temperature treatment in each well.

10.1128/mbio.03291-22.3 FIG S3 Strategy to expose strains to heat shock in a temperature gradient. (a) Scheme of the methodology utilized to generate a darkened 96-well plate with PCR tubes. The PCR tubes were externally painted with black aerosol and then sterilized with UV light for 15 min. LNN-CCD medium with luciferin (0.5 mM) was added, and then the strains were inoculated as conidial suspensions. The 96-well plate was placed in constant light (LL) at 25°C for 5 h and then transferred to DD for 12 h at 25°C. Background luciferase levels were calculated for 1 h prior to the heat shock. Luminescence was acquired with a CCD camera (indicated with bent arrows in the “Experimental setup” diagram), and tubes were exposed to heat treatment (in a gradient thermal cycler) for different times (60, 30, 15, 5, and 1 min). We continued measuring luminescence levels after the heat shock for 12 additional hours. (b) Scheme of the temperatures used for the heat shock treatment in the gradient thermal cycler in a range of 34°C to 44°C. Download FIG S3, PDF file, 0.9 MB. Copyright © 2023 Tabilo-Agurto et al. 2023 Tabilo-Agurto et al. https://creativecommons.org/licenses/by/4.0/ This content is distributed under the terms of the Creative Commons Attribution 4.0 International license.

**FIG 4:**
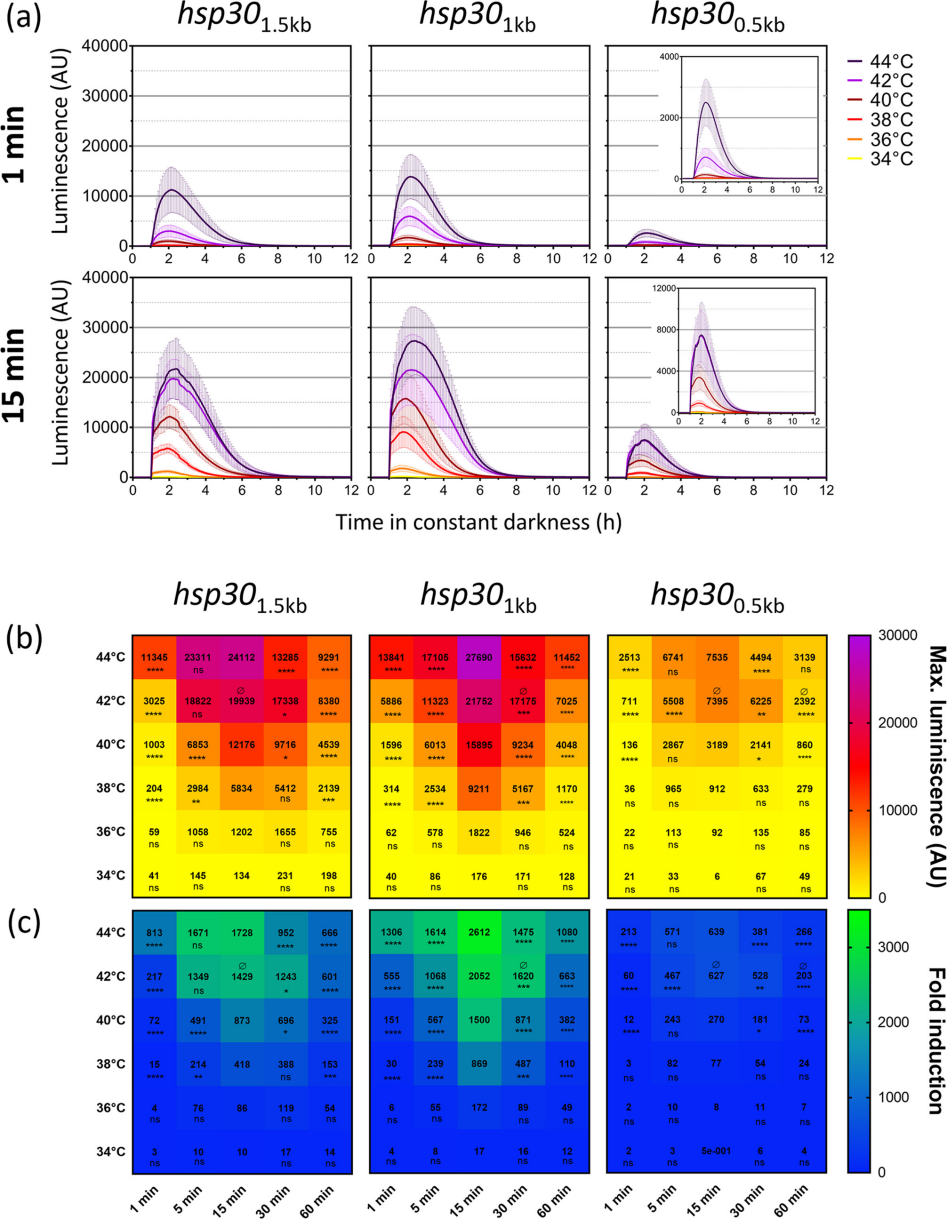
Transcriptional responses conferred by subjecting the full or resected *hsp30* promoters to a temperature gradient and different exposure times. (a) Activity profile of each *hsp30* promoter region in different temperatures after 1 and 15 min of treatment. Closeups of the *hsp30*_0.5 kb_ graphs are displayed as insets. Each curve corresponds to the average results from two biological clones with eight independent wells each ± standard deviations (SD) and represents the behavior in two independent experiments. (b and c) Maximum luminescence (b) and fold change (c) values obtained after all the heat shock treatments for each *hsp30* promoter region. The maximum luminescence was defined as the average of the highest values. The fold induction was calculated with the maximum luciferase expression (shown in panel b) with respect to the average of the background values of each promoter region before heat shock treatment. The data were obtained from the luciferase activity profiles shown in Fig. S4. Statistical significance was determined using two-way analysis of variance (ANOVA) plus Dunnett’s test (for time treatments, all values were compared to the values obtained with 15-min treatments [*, *P* < 0.05; **, *P* < 0.01; ***, *P* < 0.001; ****, *P* < 0.0001; ns, nonsignificant], and for temperature treatments, all values were compared to the values obtained with 44°C treatments [Ø, nonsignificant]). Time significance is indicated below each value, whereas for temperature, only nonsignificance (Ø) is shown above the value when it corresponds.

The *hsp30* promoters under study show that the degree of the response is augmented as temperature is progressively increased and as the duration of the stimuli is lengthened, behaving as transcriptional rheostats that tune responses upon changes in the strength of the stimuli (Fig. 4; Fig. S4). Thus, the reporters yielded increasing induction levels, reaching a maximum at 15 min, while longer heat pulses (30 and 60 min) still yielded strong responses that, in general, were lower than the peak level (15 min). Notably, a short heat pulse (1 min) was already able to elicit robust responses, although only at the highest temperatures. Thus, the *hsp30* promoter is capable of a highly tunable response even upon short pulses of heat (Fig. 4).

10.1128/mbio.03291-22.4 FIG S4 Luciferase activity profiles conferred by full or resected *hsp30* promoters using a temperature gradient and different exposure times. (a to e) Activity profiles of each *hsp30* promoter region after a short (a to b) or long (c and e) heat shock treatment. Average values and SD of each measurement are shown (2 biological clones, with eight technical replicas for each one). A closeup of the *hsp30*_0.5 kb_ graph is displayed in an inset when needed. The methodology is described in the legend to Fig. S3. Download FIG S4, PDF file, 1.5 MB. Copyright © 2023 Tabilo-Agurto et al. 2023 Tabilo-Agurto et al. https://creativecommons.org/licenses/by/4.0/ This content is distributed under the terms of the Creative Commons Attribution 4.0 International license.

The full-length promoter (*hsp30*_1.5 kb_) and the *hsp30*_1 kb_ section exhibited stronger inductions than the *hsp30*_0.5 kb_ section, reaching their maximal levels at the highest temperatures (Fig. 4b), confirming the trend seen in previous experiments (Fig. 2). Notably, the analyses revealed that the first two promoters displayed strong activation starting at 38°C, while the smallest promoter section only yielded strong transcriptional responses starting at 40°C (Fig. 4; Fig. S4). As observed in the transcriptional profiles, these *hsp30* promoters generated a graded response as temperature and treatment length variables were combined, further supporting the notion of their use as transcriptional rheostats to tune the expression of genes of interest.

To better compare the above-described data, we calculated the fold induction achieved for each promoter under different treatment conditions (Fig. 4c). This analysis confirmed that the induction attained tended to increase with higher temperatures and longer exposure times up to 15 min and that when the reporter strains were exposed for 30 or 60 min, the response was still high, although lower than the peak levels. Thus, although we initially expected to have the largest induction at the highest temperature and longest times, this was achieved instead at 15 min. These results suggest that, due to the high efficiency of thermal exchange achieved in this assay (utilizing a thermal cycler), even a lower applied temperature and a shorter exposure time suffice to generate a maximal response, whereas higher temperatures (for prolonged times) may lead to a detrimental effect on cellular function. Despite the different numbers of putative regulatory elements in the longest and the shortest promoter regions, each of the tested sections displayed a wide range of activities, confirming accurate and progressive responses to the intensity and duration of the thermal stimulus.

### Temperature-conditioned control of an N. crassa catabolic process.

With the characterization of the dynamic regulation of the *hsp30*-derived promoters, we sought to test one of them in its ability to control a gene of interest, of relevance in fungal physiology. The *hsp30*_1 kb_ region showed a response pattern similar to that of the full-length promoter, and while these promoters offer an excellent inducible system, working with long DNA segments in Neurospora can trigger repeated induced point (RIP) mutation during a sexual cross, which can lead to alteration of the endogenous copy of the DNA, as well as the additional copy (57). To minimize such problems, we decided to use the *hsp30*_0.5 kb_ section, which is closer to the ~400-bp limit at which RIP starts occurring. While choosing this smaller promoter compromises the levels of expression, it still provides a good dynamic range of regulation. Also, having a shorter promoter region makes it easier to use it in combination with other transcriptional modules.

N. crassa is known to possess great plant cell wall decomposition capabilities (58, 59), having more than 400 proteins with carbohydrate-active enzyme domains (60). Several of the genes involved in the underlying regulatory network, as well as the master controllers of the system, have been identified (2, 61). Hence, Neurospora is an excellent model organism to dissect key aspects of industrial production of second-generation bioethanol (62–65). One relevant strategy to control cellulase production is modulating the expression of one of the key genes in this pathway: the one encoding the transcription factor CLR-2 (NCU08042) (66). Thus, we aimed to command, through high temperature, the expression of *clr-2* by putting it under the control of the *hsp30*_0.5 kb_ promoter.

**FIG 5:**
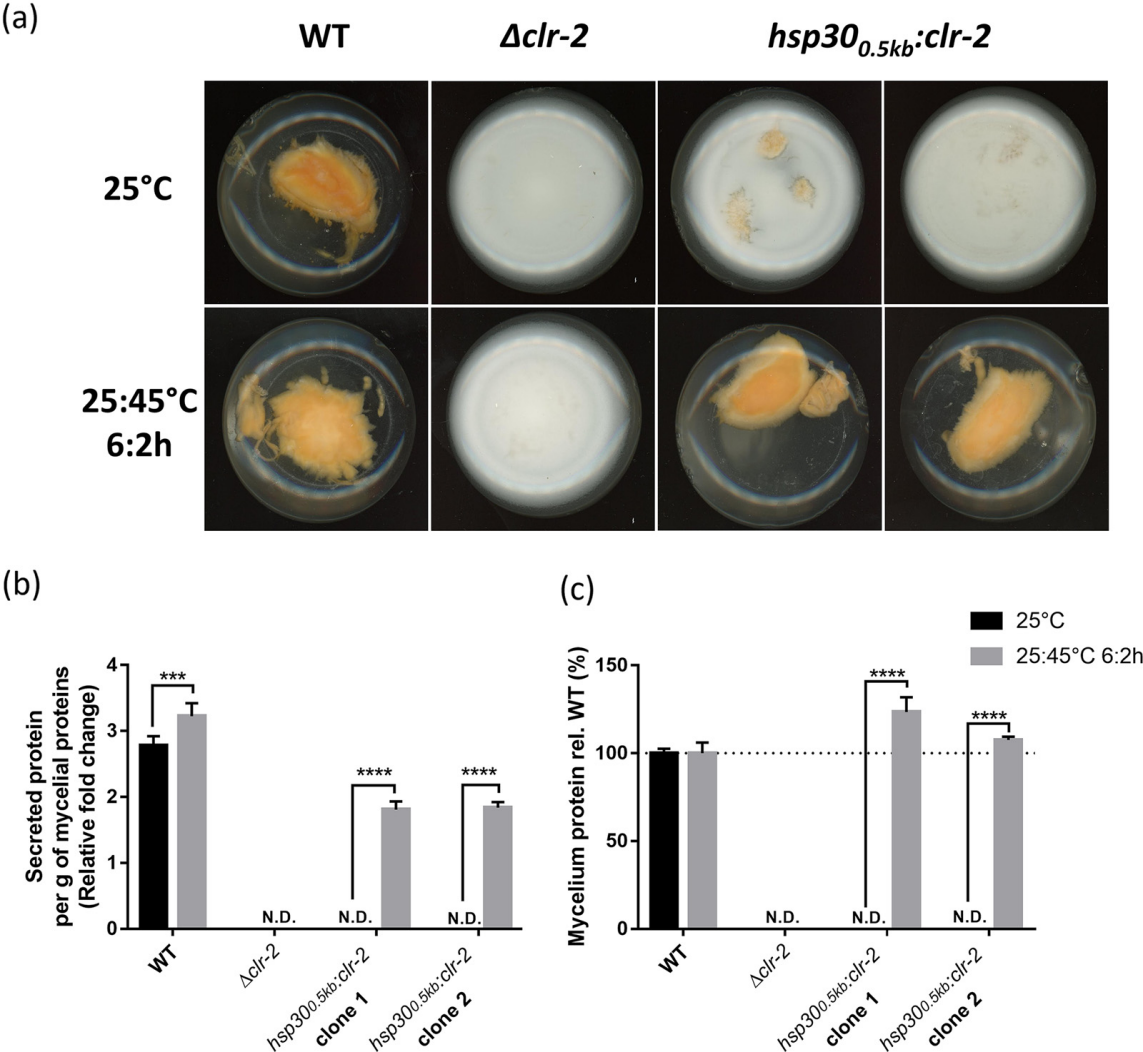
The *hsp30*_0.5 kb_ promoter can control a metabolic pathway of biotechnological interest. (a) Conidia (10^6^) from WT (x654-1), *Δclr-2*, and *hsp30*_0.5 kb_:*clr-2* (biological clones 1 and 2) strains were inoculated into Vogel’s medium with crystalline cellulose (Avicel, 2% [wt/vol]) as the carbon source. The flasks were placed under constant light conditions (LL) at 25°C with or without a high-temperature treatment (a pulse at 45°C for 2 h every 6 h [25:45°C 6:2h]). Before imaging, all the flasks were placed for 7 days in a shaker (125 rpm). The image depicts representative phenotypes from three independent experiments. (b and c) Supernatant protein concentrations (b) and total mycelial protein contents (c) were determined from 7-day cultures of WT, *Δclr-2*, and *hsp30*_0.5 kb_:*clr-2* strains grown on 2% Avicel with or without the high-temperature treatment (25:45°C 6:2h) as explained in Material and Methods. The supernatant concentrations were normalized to the total mycelial proteins per condition. The mean values and standard deviations represent three independent measurements and three independent experiments. N.D., not detected. Statistical significance was determined using two-way ANOVA plus Sidak’s test (***, *P* < 0.001; ****, *P* < 0.0001).

We evaluated the capability of the engineered strains to grow in medium with Avicel (crystalline cellulose) as the sole carbon source, knowing that *clr-2* expression is needed to induce cellulolytic gene expression and cellulose deconstruction (66). We observed that under normal temperature (25°C), the *hsp30*_0.5 kb_:*clr-2* strain failed to grow in liquid cultures containing Avicel, whereas it showed normal growth when sucrose was present instead (Fig. S5 and S6). Such lack of growth, or the negligible levels of secreted proteins of this strain in Avicel, was comparable to the results for a *Δclr-2* strain (Fig. 5; Fig. S7). Nevertheless, when Avicel cultures of *hsp30*_0.5 kb_:*clr-2* strains were exposed to daily heat shock pulses, both growth and secreted protein levels were recovered (Fig. 5; Fig. S7). On the other hand, growth on sucrose was not compromised for either type of strain (Fig. S5; Fig. S6). Thus, these results highlight the tight regulation provided by this type of promoter, and since both the intensity and frequency of the heat shock treatments can be modified, providing a broad and graded response (Fig. 4), it opens the possibility to maximize, at will, cellulase production in Neurospora, likely minimizing the fitness costs of CLR-2 overexpression (65). Notably, although the repeated application of heat shocks has a perceptible effect on mycelial growth, as measured in the wild-type (WT) strain (Fig. S8), the strong induction of a gene of interest (or in this case, the production of cellulases) may fully compensate for growth differences.

10.1128/mbio.03291-22.5 FIG S5 Phenotypic analyses of heat shock treatments in sucrose medium. Conidia (10^6^) from WT (x654-1), *Δclr-2*, and *hsp30*_0.5 kb_:*clr-2* (biological clones 1 and 2) strains were inoculated into Vogel’s medium with sucrose (2% [wt/vol]) as the carbon source. Flasks were placed in constant light (LL) at 25°C with or without a high-temperature treatment, the latter consisting of a 45°C pulse for 2 h every 6 h (25:45°C 6:2h). Cultures were kept for 7 days in a shaker (125 rpm). The photographs are representative of three independent experiments. Download FIG S5, PDF file, 1.1 MB. Copyright © 2023 Tabilo-Agurto et al. 2023 Tabilo-Agurto et al. https://creativecommons.org/licenses/by/4.0/ This content is distributed under the terms of the Creative Commons Attribution 4.0 International license.

10.1128/mbio.03291-22.6 FIG S6 The *hsp30*_0.5 kb_ promoter can control a catabolic pathway of biotechnological interest. Conidia (10^6^) from WT (x654-1), Δ*clr-2*, *hsp30*_0.5 kb_:*clr-2* (biological clones 1 and 2) were grown in Vogel’s media with sucrose (2% [wt/vol]) and crystalline cellulose (Avicel, 2% [wt/vol]) as carbon source. One set of tubes grew at 25°C, while the others were exposed to a cycle of 45°C for 2 h and 25°C for 6 h (repeated 3 times every 24 h). All tubes were placed in a shaker (125 rpm) in constant lights (LL), for 4 days (sucrose) or for 7 days (Avicel). The photographs are representative of the behavior of three independent experiments. Download FIG S6, PDF file, 1.2 MB. Copyright © 2023 Tabilo-Agurto et al. 2023 Tabilo-Agurto et al. https://creativecommons.org/licenses/by/4.0/ This content is distributed under the terms of the Creative Commons Attribution 4.0 International license.

10.1128/mbio.03291-22.7 FIG S7 Secreted protein levels in shifted cultures. (a) Conidia (10^6^) from WT (x654-1), *Δclr-2*, and *hsp30*_0.5 kb_:*clr-2* (biological clones 1 and 2) strains were inoculated into Vogel’s medium with sucrose and were grown under constant light conditions (LL) at 25°C for 48 h. Then, mycelia were washed and transferred to Vogel’s medium with crystalline cellulose (Avicel, 2% [wt/vol]) as the carbon source, and the flasks were placed under constant light conditions (LL) at 25°C with or without a high-temperature treatment (a pulse at 45°C for 2 h every 6 h [25:45°C 6:2h]). Supernatant protein concentrations were determined from 24 h cultures of the WT, *Δclr-2*, and *hsp30*_0.5 kb_:*clr-2* strains grown on 2% Avicel with or without the high-temperature treatment (25:45°C 6:2h) as explained in Material and Methods. The mean values and standard deviations represent three independent measurements in three independent experiments. Statistical significance was determined using two-way ANOVA plus Sidak’s test (***, *P* < 0.001; ****, *P* < 0.0001). Download FIG S7, PDF file, 0.1 MB. Copyright © 2023 Tabilo-Agurto et al. 2023 Tabilo-Agurto et al. https://creativecommons.org/licenses/by/4.0/ This content is distributed under the terms of the Creative Commons Attribution 4.0 International license.

10.1128/mbio.03291-22.8 FIG S8 Effect of high-temperature treatment on WT growth. Conidia (10^6^) from the WT (x654-1) were inoculated into Vogel’s medium with sucrose (2% [wt/vol]) as the carbon source. Flasks were placed in constant light (LL) at 25°C with or without high-temperature treatment (a pulse at 45°C for 2 h every 6 h [25:45°C 6:2h]). Cultures were kept for 4 days in a shaker (125 rpm), and then the mycelium was harvested and dried. Statistical significance was determined by the Student *t* test (****, *P* < 0.0001). Download FIG S8, PDF file, 0.1 MB. Copyright © 2023 Tabilo-Agurto et al. 2023 Tabilo-Agurto et al. https://creativecommons.org/licenses/by/4.0/ This content is distributed under the terms of the Creative Commons Attribution 4.0 International license.

### Design of synthetic promoters derived from *hsp30* putative *cis* elements.

**FIG 6:**
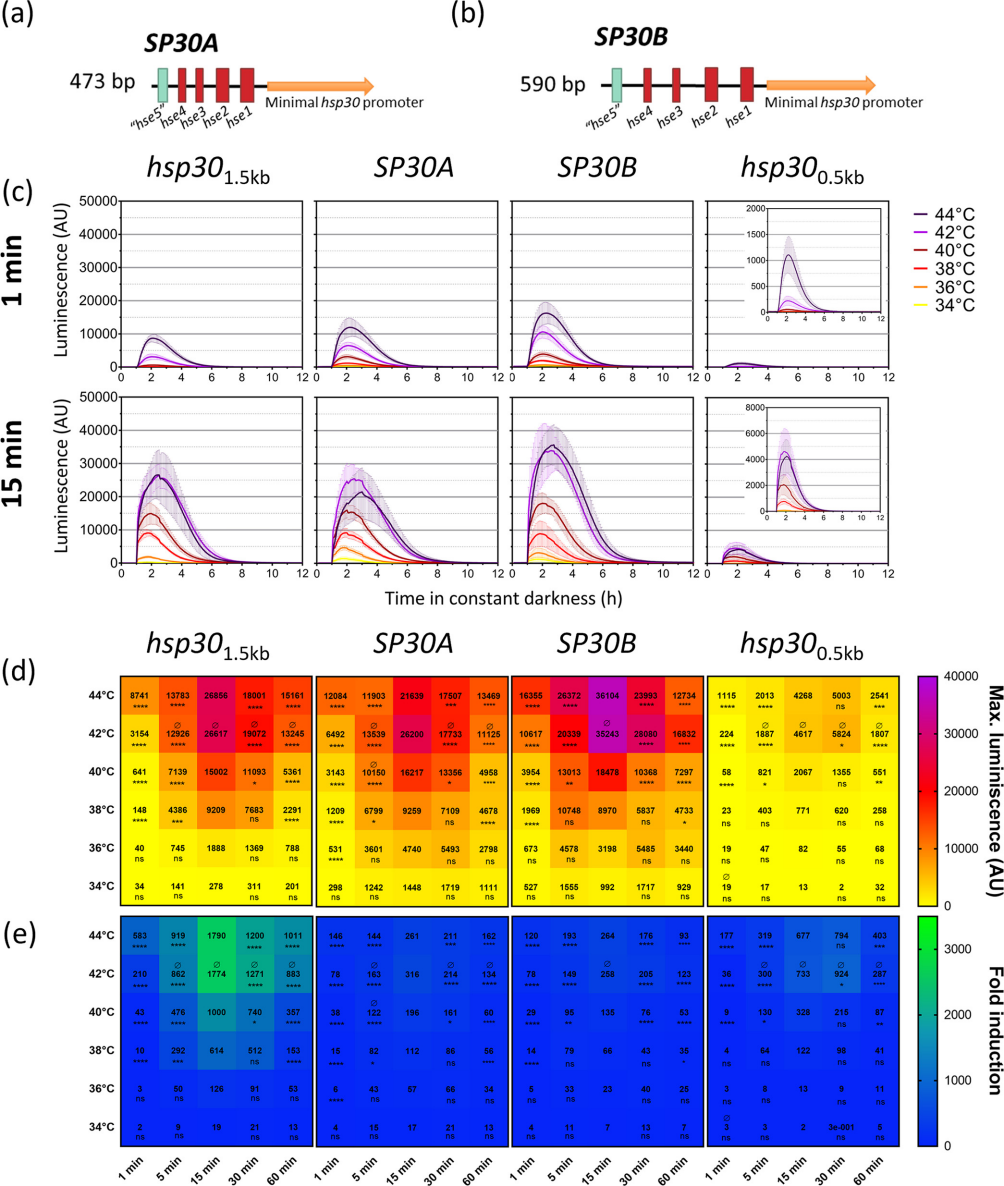
Design of two inducible synthetic promoters tunable by subtle changes in heat shock treatments. (a and b) Schemes of *hsp30* synthetic promoters (*SP30*), where synthetic promoters with 24-bp (*SP30A*) (a) or 50-bp (*SP30B*) (b) spacers between the indicated putative *hse* were used to generate reporter genes in the context of a minimal *hsp30* promoter of 250 bp. (c) Luciferase activity profiles of the synthetic promoters against a temperature gradient for 1 min and 15 min. Closeups of the *hsp30*_0.5 kb_ graphs are displayed as insets. Each curve corresponds to the average values from two or three biological clones with four independent wells each ± standard deviations (SD) and represents the behavior in two independent experiments. (b and c) Maximum luminescence (b) and fold change (c) values observed after the heat shock treatments for the indicated *hsp30* promoters are indicated. The maximum luminescence was defined as the average of the highest values. Fold induction was calculated based on the maximum luciferase expression (shown in panel b) with respect to the average of the background values of each promoter region before heat shock treatment. The data were obtained from the luciferase activity profiles shown in Fig. S10. Statistical significance was determined using two-way ANOVA plus Dunnett’s test (for time treatments, all values were compared to the values obtained with 15-min treatments [*, *P* < 0.05; **, *P* < 0.01; ***, *P* < 0.001; ****, *P* < 0.0001; ns = non-significant], and for temperature treatments, all values were compared to the values obtained with 44°C treatments [Ø, nonsignificant]). Time significance is indicated below each value, whereas for temperature, only nonsignificance (Ø) is shown above the value when it corresponds.

To generate a versatile molecular tool to be used in N. crassa where we could combine the strength and tunable aspects of *hsp30* promoters in a modular fashion while also reducing the incidence rate of RIP-based mutations, we proceeded to design *hsp30*-based synthetic promoters (*SP30*) using the putative *hse* sequences present in the *hsp30* regulatory region. For this, we selected the predicted minimal promoter region of *hsp30* (up to −250 bp) and added to it the putative *hse* sequences. In addition to the four predicted *hse* in the *hsp30* promoter (Fig. 1a), we identified, through bioinformatics tools (see Materials and Methods), a fifth *hse* element in *hsp30* (Table S1 at https://doi.org/10.6084/m9.figshare.21774671 and Table S2 at https://doi.org/10.6084/m9.figshare.21774632), with *P* values similar to those of the putative *hse1* and *hse2* sequences, which are the most conserved in *hsp30*. Thus, we designed two short versions of these *hsp30* synthetic promoters: one with 24-bp spacers between the five *hse* (*SP30A*) (Fig. 6a; Fig. S9), and another one with 50-bp spacers between the five *hse* (*SP30B*) (Fig. 6b; Fig. S9). The synthetic promoters were evaluated *in vivo* by using luciferase reporters to analyze their expression profiles over a temperature gradient during long (60 min) and short (1 min) heat pulses (Fig. S10). As we envisioned, the two synthetic promoters displayed strong responses to heat shock treatments, showing tunability to variation in both temperature and exposure times (Fig. 6c and d).

10.1128/mbio.03291-22.9 FIG S9 FASTA sequences of the *SP30A* and *SP30B* promoters. The sequences of both synthetic promoters (*SP30A* and *SP30B*). *hse* (red), the newly assigned *hse* (light blue), spacers (black), and the putative minimal promoter of *hsp30* (deep red) are indicated. Download FIG S9, PDF file, 0.2 MB. Copyright © 2023 Tabilo-Agurto et al. 2023 Tabilo-Agurto et al. https://creativecommons.org/licenses/by/4.0/ This content is distributed under the terms of the Creative Commons Attribution 4.0 International license.

10.1128/mbio.03291-22.10 FIG S10 Luciferase activity profiles conferred by *SP30A* and *SP30B* using a temperature gradient and different exposure times. (a to e) Activity profiles of *SP30A*, *SP30B*, *hsp30*_1.5 kb_, and *hsp30*_0.5 kb_ promoters after a short (a to b) or long (c and e) heat shock treatment. The average value and SD of each measurement are shown (2 to 3 biological clones, with four technical replicas for each one). A closeup of the *hsp30*_0.5 kb_ graph is displayed in an inset when needed. The methodology is described in the legend to Fig. S3. Download FIG S10, PDF file, 1.4 MB. Copyright © 2023 Tabilo-Agurto et al. 2023 Tabilo-Agurto et al. https://creativecommons.org/licenses/by/4.0/ This content is distributed under the terms of the Creative Commons Attribution 4.0 International license.

While the native *hsp30* promoter had an outstanding on/off ratio, given in part by the extremely low basal levels of expression, the synthetic promoters displayed higher basal activity, which negatively affected this relationship (Fig. 6e; Fig. S11 at https://doi.org/10.6084/m9.figshare.21774653). Nevertheless, the basal levels of these promoters were considerably lower than those of *hsp70* and *hsp80* (Fig. 2; Fig. S1). Both synthetic promoters behaved similarly, exhibiting (across all the temperatures/exposure times) maximal activities equivalent to or higher than that of the native *hsp30* system (Fig. S10). Despite this high activity upon induction, due to their increased basal expression, they yielded smaller fold inductions than *hsp30*_0.5 kb_ (Fig. S12 at https://doi.org/10.6084/m9.figshare.21774680) .

Thus, these synthetic *hsp30* promoters represent a viable strategy to provide a heat shock response of genes of interest in a modular fashion and conserving the key characteristics of the native *hsp30* system regarding tunability and maximum activity.

### Discussion.

Changes in temperature are a ubiquitous physical stimulus to which all organisms are exposed, and consequently, they have developed mechanisms that help them cope with strong increases in temperature, such as heat shocks. These mechanisms involve heat shock proteins (HSPs) and their accurate transcriptional regulation, which play a relevant role in high-temperature tolerance. In addition to their protective function, it has been possible to use *hsp* promoters as molecular tools for spatial-temporal control of genes of interest in several organisms (39, 67–69), a strategy that has been poorly exploited in filamentous fungi. In this work, we profiled the response of three N. crassa *hsp* promoters (and their resected versions) to discrete changes in temperature for different treatment durations to obtain a better understanding of the dynamic biological response of such genes. Moreover, we aimed to select the most suitable candidate to be used as an inducible system that, more than just an on/off switch, could act as a rheostat to provide graded transcriptional responses, depending on the intensity of the physical stimuli.

All the Neurospora promoters (*hsp30*, *hsp70*, and *hsp80*) characterized herein showed a rapid and strong response under standard heat shock treatment of 45°C for 1 h (70) in an incubator, with *hsp30*_1.5 kb_ having the highest response. It is known that low-molecular-weight HSPs tend to be the first ones to act against protein denaturation at high temperatures, due to their inability to bind and hydrolyze ATP like other, larger HSPs, and that they would thus have a strong response to heat stress (71). Therefore, the higher response levels of the *hsp30* promoter appear to correlate with their described role upon heat stress. The expression levels observed under noninducing conditions in this work revealed a basal constitutive expression of the *hsp70* and *hsp80* genes, in contrast to *hsp30*, which goes from extremely low expression levels to strong induction in response to thermal stimulation. Previous investigations reported that *hsp30* mRNA levels are rather negligible at normal growth temperatures (72), while high basal expression is characteristic of the HSP members of the HSP70 and HSP90 families (73).

The resected versions of these promoters provided information on the possible roles of their relevant *cis* elements. In *hsp70*, these elements are located in the *hsp70*_0.6 kb_ region, and the absence of a further upstream sequence did not cause a decrease in the thermal response. On the contrary, the relevant regulatory elements in *hsp80* appeared to be upstream from the *hsp80*_0.6 kb_ region, because this resected section failed to show induction despite having the majority of the proposed *tre*-like boxes. In contrast, the *hsp30*_1 kb_ region had the same tunability and responsiveness as *hsp30*_1.5 kb_, suggesting that several of the relevant elements were already present in this resected region. On the other hand, the presence of only two of the four proposed *hse* in *hsp30*_0.5 kb_ may explain its strong activity at 45°C but diminished responses at a temperature closer to 35°C, as it has been reported that having multiple *hse* tends to confer higher response levels (73, 74).

Our data indicate that the *hsp30* sections analyzed display key features of an ideal regulable promoter, allowing progressive control of transcription at different stimulus intensities. They also provide tight regulation that can maintain low basal levels of expression in the absence of thermal stimulation, in addition to a rheostat-like behavior (high tunability) and temporal control (75). The *qa-2* and *vvd* promoters are probably some of the most used inducible promoters in Neurospora; however, *qa-2* has a limited range of expression and a low, albeit rather leaky basal transcription in the absence of quinic acid (21). It also has the additional caveat that, once the inducer is added, it is cumbersome to remove it, as it would require medium exchange. Overall, the *vvd* and *hsp30* promoters share key properties; for example, there is no need to supplement the growth medium with an inducer as they both respond to a physical stimulus that can be externally and easily provided (75). However, the particular light/dark condition requirements for controlling *vvd* (18) imply defined laboratory settings (dark room), and its use may not be fully compatible with photobiology or circadian studies.

Thus, the *hsp30* system described here shows flexibility, as the response can be fine-tuned not only by the inducing temperature but also by the length of the treatment (Fig. 4; Fig. S4). This was clearly exemplified in the discrete changes in temperature provided by the thermal cycler approach, where the selected reporter strains were treated at six different high temperatures simultaneously. This simple experimental setup for heat shock treatment provides several advantages, including the following: (i) it allows the exposure of the strains to a wide range of temperatures, (ii) optimizes the time required between experiments, and (iii) increases the precision and accuracy of the temperature at which the strains are exposed (particularly as PCR tubes are designed for efficient heat transmission). Although we were able to obtain an accurate profile of the Neurospora *hsp30* promoter responses with this setup, the limitations of the methodology used should also be noted. The activity profiles at high temperatures for prolonged treatments (60 min and 30 min) can be weaker than the ones obtained at shorter times, which is probably caused by the efficient temperature transfer in the thermal cycler compared to the one inside an incubator, where the former might result in high stress at longer time points, compromising cellular status and impairing reporter output. In addition, the use of a 96-well format (PCR tubes) limits the amounts of medium that can be utilized, restricting growth as well. In our hands, the tested strains grew well for at least 2 full days.

The map of *hsp30* responses to different temperatures shows a particular pattern: at short exposure times, luciferase expression mostly correlates with the increasing temperatures, and there is also a clear gradient of responses between 15-min and 1-min treatments (Fig. 4b; Fig. S4). Nevertheless, at longer exposure times, a diminished response is generally observed if compared to the responses with 15-min exposures. As mentioned earlier, this could be attributed to excessive stress caused by the direct and prolonged exposure to high temperatures, which could also explain the augmented dispersion of the data obtained at such temperatures and exposure times. Despite this, we could observe a wide range of response intensities depending on the degree of the heat shock and the duration of this stress for the *hsp30* promoters (Fig. 4). Importantly, the data obtained herein reveal that the latter are highly modulable in a range of high temperatures, exhibiting also an analogue (gradual and continuous) and not digital/binary (on/off) (76) response.

As proof of principle, we conditioned Neurospora cellulolytic capabilities by putting *clr-2* expression under the control of the *hsp30*_0.5 kb_ promoter. Notably, although the resected promoter that we used had lower activity levels than the longer *hsp30* sections, the results clearly demonstrate how our system is capable of tightly regulating a gene of interest that can have ecophysiological implications (77) and industrial impact (78). A main obstacle in the industrial application of the degradation of cellulose has been the high cost of enzyme production, which has restricted accessing lower prices of bioethanol as a fuel alternative, a discussion that is revived every time gas prices are on the rise (79, 80). Using an *hsp30*_0.5 kb_ promoter and temperature treatments to regulate *clr-2* expression, we reverted the poor growth phenotype in cellulose (equivalent to the one seen in a *Δclr-2* strain), although the secreted proteins levels were lower than those of a WT strain (Fig. 5). Nevertheless, when we used a different protocol, consisting of transferring sucrose-grown mycelia to Avicel, we observed low levels of protein secretion for the *hsp30*_0.5 kb_:*clr-2* strains at 25°C, a situation that was reverted—surpassing even the WT levels—when heat pulses were applied (Fig. S7). In addition, other induction protocols (with more frequent heat shocks) or the implementation of other *hsp30* promoter synthetic versions could easily yield augmented cellulase levels. While further analyses are necessary to advance and optimize this methodology, the thermal induction strategy described herein presents itself as an attractive alternative to regulate the expression of genes of interest and to tightly regulate and tune desired phenotypes.

Furthermore, to facilitate the adoption of an *hsp30*-based system, we designed a synthetic version of *hsp30*, for which we utilized the *hsp30*_1.5 kb_ promoter’s putative *hse*. Despite the lack of nucleotide resolution studies characterizing these *hse* as functional, their sequence identity strongly suggests that these regions are conserved and are likely recognized by HSFs, commanded by the major regulator in Neurospora, HSF-1 (NCU08512). In addition, it has been shown that the HSF of Neurospora can efficiently recognize an *hse* from yeast (81). Based on this, we used one of the HSF-1 motifs obtained from yeast and N. crassa to further identify new *hse* and confirm the previously proposed *hse* in the *hsp30* promoter (Table S1 at https://doi.org/10.6084/m9.figshare.21774671 and Table S2 at https://doi.org/10.6084/m9.figshare.21774632). Thus, we detected a new *hse* (*hse5*) that may also play a part in the *hsp30*_1 kb_ regulation. With this information, we designed two synthetic promoters based on the five putative *hse* present in *hsp30*, where we contemplated having different lengths of spacers between the *hse*. It has been observed that the optimal spacer between the *cis* elements can maximize response, although such levels would depend on the promoter/organismal context. In bacteria, the optimal distance between the minimal *cis* elements in the core promoter is 17 bp for E. coli (82) but reaches greater lengths of up to 80 bp in the case of Pseudomonas (83), whereas in eukaryotes, the minimal distances between the TATA box and the transcriptional start site (tss) are ~30 bp, observed in yeast (84) and mammals (85).

We were able to generate a high range of tunability, although the low background expression of the native *hsp30* promoter was not fully maintained (Fig. 6; Fig. S11 at https://doi.org/10.6084/m9.figshare.21774653). Indeed, issues like this can sometimes be a trade-off for synthetic promoters, where despite strong responses, basal expression is higher than expected (86). Nevertheless, reproducibility, tunability, and temporal controllability are properties that are still present in the designed synthetic *hsp30* promoters. In addition, the high conservation in the regulation of *hsp* expression allows the prediction that some of these resected promoters could readily work in other ascomycetes (Fig. S13 at https://doi.org/10.6084/m9.figshare.21774677). In particular, it will be interesting to attest the behavior of the modular synthetic *hsp30* promoters in biotechnologically relevant fungi like Aspergillus niger or Trichoderma reesei.

Thus, in this work we provided a detailed profile of the response of the *hsp* genes to thermal stimuli, while also extending the molecular tools available for N. crassa by describing a new set of inducible heat shock promoters that have overall low background levels and allow a rheostat-like adjustment of the expression of a gene of interest.

### Materials and methods: plasmid construction.

All the plasmids were assembled by yeast recombinational cloning (87) in Saccharomyces cerevisiae strain BY4741 (*MATa his3Δ1 leu2Δ0 met15Δ0 ura3Δ0*), using promoter fragments amplified from WT (74A) N. crassa genomic DNA. For the synthetic promoters *SP30A* and *SP30B*, the fragments were synthesized by Genewiz (https://www.genewiz.com/) and then cloned as described above. The information about backbone plasmids, PCR products, and primers used for each construction is detailed in Table S4 at https://doi.org/10.6084/m9.figshare.21774650. All the constructions generated were confirmed by sequencing.

### Strains and culture conditions.

The transcriptional reporter strains, which contained the analyzed promoters (full, resected, and synthetic) fused to a destabilized luciferase (*lucPEST*) and targeted to the *csr-1* locus for cyclosporine selection (14), were transformed into a selected strain (x654-1; *ras1^bd^ mus51^RIP^ a*) as previously reported (88), following a standard electroporation protocol (11).

The *Δclr-2* strain (xc2386-2; *Δclr-2 ras1^bd^ mus51^RIP^ a*) was obtained from a cross between #15834 (A) and L418T654c-1 (*ras1^bd^ mus51^RIP^ a*). The *hsp30*_0.5 kb_:*clr-2* (xc2417; *ras-1^bd^ mus51^RIP^*) strains used for cellulose phenotypic assays were obtained by replacing the 2,000-bp upstream region (relative to the ORF) of the *clr-2* gene (NCU08042) with the *hsp30*_0.5 kb_ promoter in an x654-1 background (primers are listed in Table S4 at https://doi.org/10.6084/m9.figshare.21774650). The selection was made by incorporating a *bar* resistance cassette upstream from the resected promoter, and homokaryotic strains were obtained through sexual crosses (89).

The vegetative growth utilized slants with 1× Vogel minimal medium (VM) (90) supplemented with 2% (wt/vol) sucrose in 1.5% (wt/vol) agar for 5 to 7 days in constant light (LL) at 25°C, whereas for sexual crosses, synthetic crossing medium (SCM) (89) was used. Sorbose-containing medium (FIGS) was utilized for colony isolation and ascospore germination (91). Ascospores were picked on slants containing VM medium supplemented with bialaphos (92), cyclosporine (5 μg/mL), and/or luciferin (10 μM), in order to select progeny carrying knockout cassettes and/or reporter activity. To conduct luciferase analyses in both heat shock treatment setups (see below), we used LNN-CCD medium (0.03% glucose, 0.05% arginine, 50 ng/mL biotin, 1.5% agar) (93) supplemented with the indicated concentrations of luciferin.

### Heat shock analysis.

The heat shock treatments were conducted by applying two different strategies: the first using an incubator for exploratory functional analyses and the second using a gradient thermal cycler for high-throughput and more accurate studies, as follows.

(i) For the incubator strategy, the strains were grown in black, 96-well cell culture plates for 24 h in LL at 25°C with 150 μL of LNN-CCD medium and luciferin (0.5 mM) per well, and the plates were covered with a breathable transparent membrane. After 24 h of monitoring Luc activity, a temperature pulse was provided during 1 h of treatment, utilizing a Percival incubator. The temperatures chosen for this exploratory analysis were 30°C, 35°C, and 45°C.

(ii) For the thermal cycler strategy, the strains were inoculated into 96-well plates made of PCR tubes, covered on the outside with black spray paint resistant to temperature (Rust-Oleum) to avoid light cross-contamination and covered with a breathable transparent membrane. Strains were grown for 5 h in LL plus 12 h under constant darkness conditions (DD) at 25°C, using 50 μL of LNN-CCD medium and luciferin (0.5 mM). After that, the Luc activity was monitored for 1 h to then apply a temperature treatment by exposing the strains to a temperature gradient (34°C, 36°C, 38°C, 40°C, 42°C, and 44°C) with varying treatment times (60 min, 30 min, 15 min, 5 min, or 1 min) in a Veriti thermal cycler (product number 4375786; Applied Biosystems) to finally record the Luc activity for the following 12 h. A scheme of this heat shock treatment methodology can be found in Fig. S3. Importantly, the hot lid of the equipment was manually disengaged in order to eliminate additional sources of heat that could interfere with the induction protocol.

Both strategies, then, involved the use of Percival incubators equipped with charge-coupled-device (CCD) Pixis 1024B cameras (Princeton Instruments) to register the luciferase expression using acquisition settings of 5 min of exposure and 3 or 12 frames per hour for the incubator strategy and the thermal cycler strategy, respectively (93).

### Comparison of Neurospora crassa inducible promoters.

We compared, using luciferase transcriptional reporters, *hsp30* with the well-known light-inducible promoter *vvd* (NCU03967; 3.5-kb upstream region) and the quinic acid (QA)-inducible promoter of *qa-2* (NCU06023; 600-bp upstream region). The primers and plasmids used to generate these constructions are detailed in Table S4 at https://doi.org/10.6084/m9.figshare.21774650.

The analyses were conducted in 96-well deep well cell plates and PCR tubes using LNN-CCD medium with luciferin as detailed above. To compare light, temperature, and QA induction, strains were grown overnight at 25°C in LL, and then Luc activity was monitored at 25°C in DD. After 5 h, strains were subjected to the corresponding treatment, as follows, after which Luc activity was immediately monitored: (i) light pulse, 1 h of white light at 25°C (100 μM/m^2^/s; wavelength, 400 to 720 nm); (ii) temperature pulse, 1 h in DD at 45°C (depending on the experimental setup, the heat pulse was given using a thermal cycler or an incubator as indicated above); and (iii) quinic acid, a drop of 1 M QA added to each 96-well tube to obtain a final concentration of 0.01 M.

### Growth on cellulose phenotypic assays.

Flasks containing 50 mL of minimal Vogel’s 1× medium supplemented with 2% (wt/vol) sucrose or Avicel were inoculated with conidial suspensions. Strains were grown for 7 days with agitation in Percival incubators in LL, where control strains were kept at 25°C and strains subjected to temperature treatments received a temperature pulse of 2 h at 45°C every 8 h (three times a day) for 7 days.

Additionally, flasks containing 50 mL of minimal Vogel’s 1× medium supplemented with 2% (wt/vol) sucrose were inoculated with conidial suspensions. Strains were grown for 48 h with agitation in Percival incubators in LL, and then mycelia were washed and transferred to flasks containing 50 mL of minimal Vogel’s 1× medium supplemented with 2% (wt/vol) Avicel, where control strains were kept at 25°C and strains subjected to temperature treatments received a temperature pulse of 2 h at 45°C every 8 h (three times a day) for 24 h.

Biomass and protein quantification of each condition was performed using the dry weight of grown mycelium and Bradford curve interpolation, respectively, as previously described (9).

### Heat shock element (*hse*) sequence analysis.

We used the YeTFaSCo database (http://yetfasco.ccbr.utoronto.ca/index.php) for S. cerevisiae HSF-1-based motifs. The motif with identification number (ID) 615 was used as a matrix to identify new *hse* or to confirm the previously described *hse* in the *hsp30* promoter using FIMO of MEME suite (version 5.4.1). A *P* value of <0.001 was used to select the *hse*. The results of this analysis can be found in Table S1 at https://doi.org/10.6084/m9.figshare.21774671.

For *hse* identification in the *hsp30* promoter through the HSF-1 motif of N. crassa, we used the CIS-BP database (http://cisbp.ccbr.utoronto.ca/) (94). The motif with ID T243453 (HSF-1, NCU08512) was utilized to analyze the *hsp30* promoter sequence using the tool “Scan single sequences for TF binding” (motif model: PWMs-LogOdds). The default threshold was utilized (score not under 8). The results of this analysis can be found in Table S2 at https://doi.org/10.6084/m9.figshare.21774632.

For the *hse* alignments, the promoters of *hsp30* in fungal orthologs (N. crassa NCU09364, Neurospora tetrasperma NEUTE1DRAFT_72918, Neurospora discreta NEUDI_159228, T. reesei TRIREDRAFT_122363, A. niger M747DRAFT_254277, Aspergillus nidulans AN2530, and Aspergillus fumigatus Afu3g14540) were all downloaded from FungiDB, and the DNA sequence alignment was performed with MEGA version 11 software with the ClustalW algorithm (95). The putative *hse* sequences were sought individually at each promoter, and then, with these sequences, multiple nucleotide sequence alignments were performed as indicated above.

### Statistical analysis.

Graphs and statistical analyses were made using GraphPad (Prism) version 7.0.
