# Supplementary material for: Developing a Temperature-Inducible Transcriptional Rheostat in Neurospora crassa
Source: mBio. 2023 Feb 6;14(1):e03291-22. doi: 10.1128/mbio.03291-22 (PMC9973361; doi:10.1128/mbio.03291-22)
Supplement: FIG S1 [file mbio.03291-22-s0001.pdf]

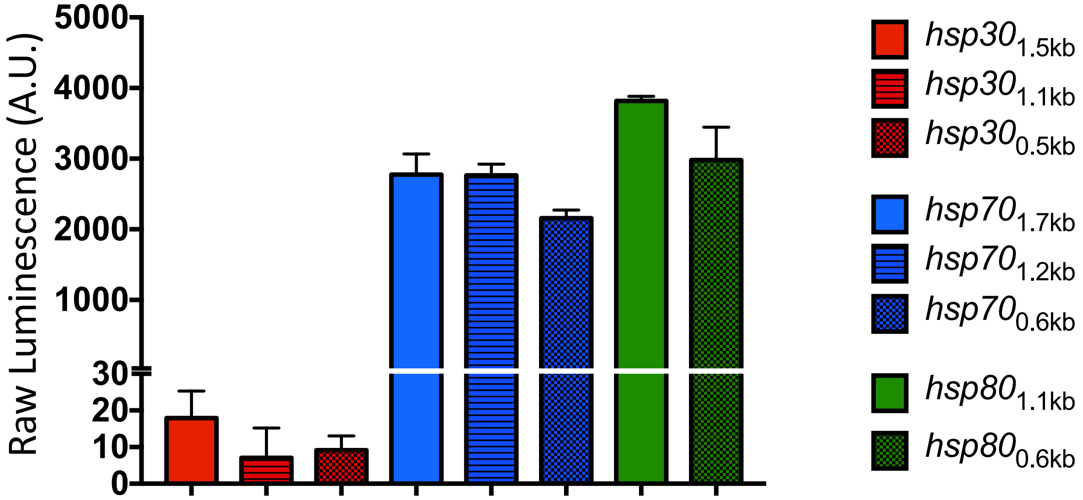

Figure S1. Basal activity of *hsp* reporters. Basal (background) luminescence levels of each *hsp* promoter and their resected sections. Each bar indicates the average of two or three biological clones with four independent wells each ± standard deviation (SD), and represents the behavior of two independent experiments. Values were obtained prior to delivering the heat shocks, in a 96-well plate format (values come from Figure 2 datasets).
