# Supplementary material for: Developing a Temperature-Inducible Transcriptional Rheostat in Neurospora crassa
Source: mBio. 2023 Feb 6;14(1):e03291-22. doi: 10.1128/mbio.03291-22 (PMC9973361; doi:10.1128/mbio.03291-22)
Supplement: FIG S2 [file mbio.03291-22-s0002.pdf]

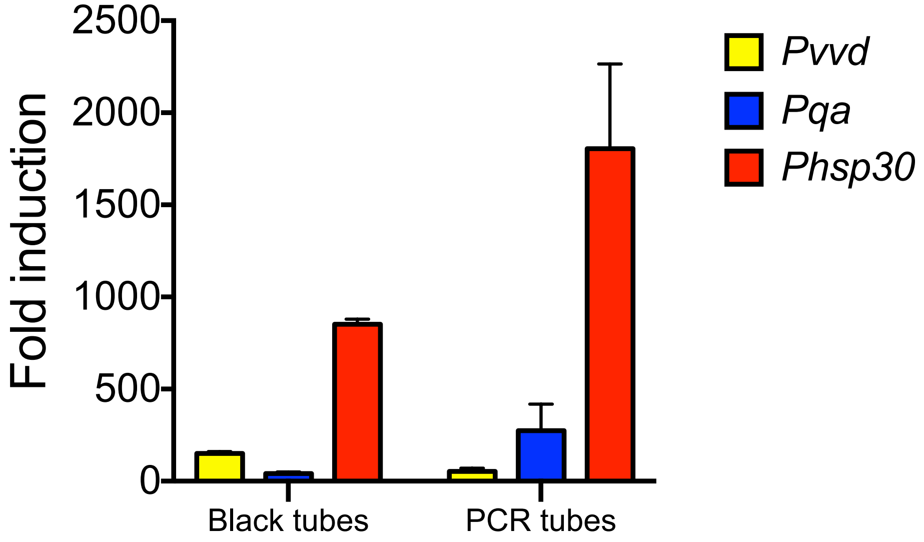

Figure S2. Comparison of fold-changes achieved by *hsp30* and other inducible *N. crassa* promoters. Fold induction achieved by the *hsp30, vvd* and *qa (qa-2)* promoters after providing the respective cognate stimuli. Fold induction was calculated with the maximum luciferase expression respect to the average of the background values before inducing each promoter.
