# Supplementary material for: Developing a Temperature-Inducible Transcriptional Rheostat in Neurospora crassa
Source: mBio. 2023 Feb 6;14(1):e03291-22. doi: 10.1128/mbio.03291-22 (PMC9973361; doi:10.1128/mbio.03291-22)
Supplement: FIG S3 [file mbio.03291-22-s0003.pdf]

(a)

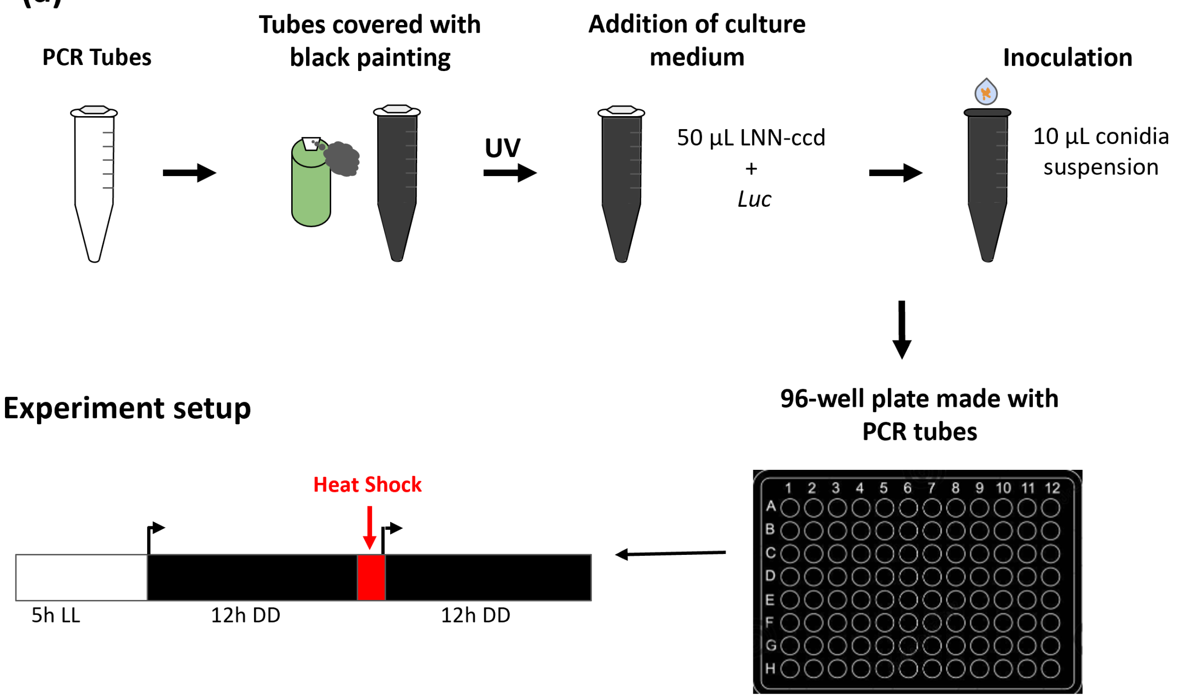

(b)

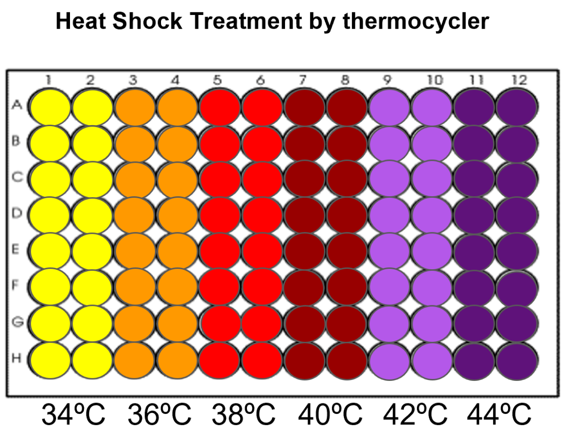

Figure S3. Strategy to expose strains to heat shock in a temperature gradient. (**a**) Scheme of the methodology utilized to generate a darkened 96-well plate with PCR tubes. The PCR tubes were externally painted with black aerosol, and then sterilized with UV light for 15 min. LNNccd media with luciferin (0.5 mM) was added, and then the strains were inoculated as conidia suspensions. The 96-well plate was placed in constant light (LL) at 25ºC for 5 h, and then transferred to DD for 12h at 25ºC. Background luciferase levels were calculated for 1 hour prior to the heat-shock. Luminescence was acquired with a CCD camera (indicated with broken arrows in the "Experimental setup" diagram), and tubes were exposed to heat treatment (in a gradient thermocycler) for different times (60, 30, 15, 5, and 1 min). We continued measuring luminescence levels after the heat shock for 12 additional hours. (**b**) Scheme of the temperatures used for the heat- shock treatment in the gradient thermocycler in a range of 34ºC to 44ºC.
