# Supplementary material for: Developing a Temperature-Inducible Transcriptional Rheostat in Neurospora crassa
Source: mBio. 2023 Feb 6;14(1):e03291-22. doi: 10.1128/mbio.03291-22 (PMC9973361; doi:10.1128/mbio.03291-22)
Supplement: FIG S4 [file mbio.03291-22-s0004.pdf]

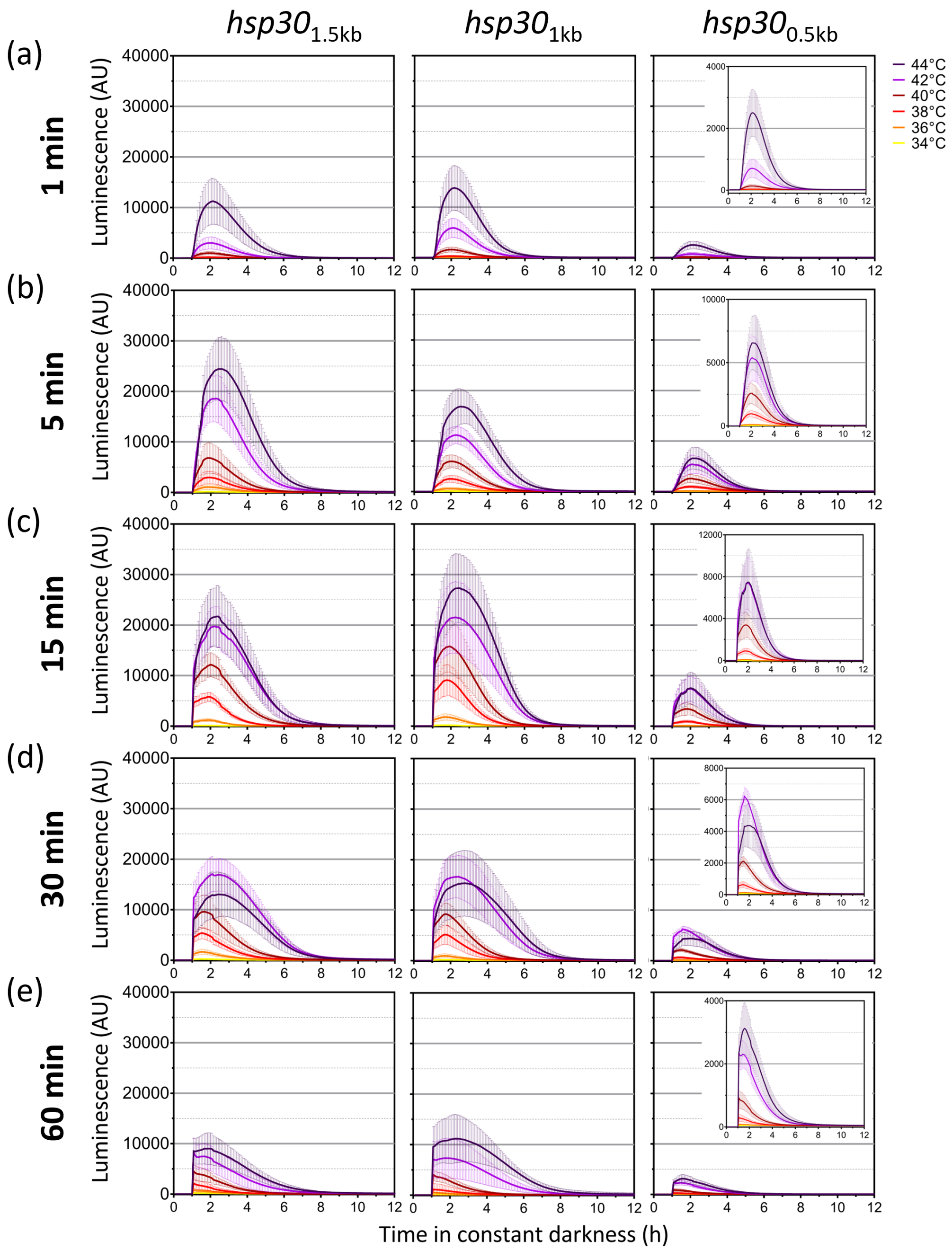

Figure S4. Luciferase activity profiles conferred by full or resected *hsp30* promoters to a temperature gradient and different exposure times. (**a** to **e**) Activity profiles of each *hsp30* promoter region after a short (**a** to **b**) or long (**c** and **e**) heat shock treatment. Average and SD of each measurement are shown (2 biological clones, with eight technical replicas for each one). A close-up of the $hsp30_{0.5kb}$ graph is displayed up on the right side when needed. The methodology is described in Figure S2.
