# Supplementary material for: Developing a Temperature-Inducible Transcriptional Rheostat in Neurospora crassa
Source: mBio. 2023 Feb 6;14(1):e03291-22. doi: 10.1128/mbio.03291-22 (PMC9973361; doi:10.1128/mbio.03291-22)
Supplement: FIG S5 [file mbio.03291-22-s0005.pdf]

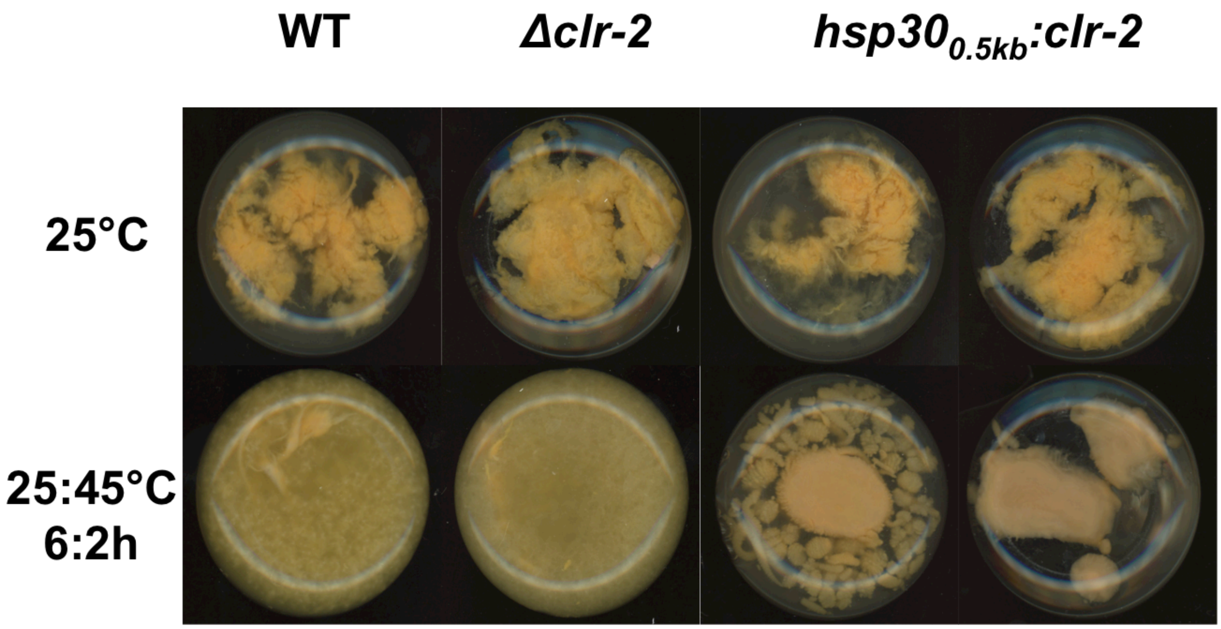

Figure S5. Phenotypic analyses of heat shock treatments in sucrose media. Conidia (10^6) from WT (x654-1), *Δclr-2*, *hsp30*$_{0.5kb}$:*clr-2* (biological clones 1 and 2) were inoculated in Vogel's media with sucrose (2%w/v) as carbon source. Flasks were grown in constant lights (LL) at 25°C with or without a high-temperature treatment, the latter corresponding of a 45°C pulse for 2 h every 6 h (25:45°C 6:2 h). Cultures were kept for 7 days in a shaker (125 rpm). The photographs are representative of three independent experiments.
