# Supplementary material for: Developing a Temperature-Inducible Transcriptional Rheostat in Neurospora crassa
Source: mBio. 2023 Feb 6;14(1):e03291-22. doi: 10.1128/mbio.03291-22 (PMC9973361; doi:10.1128/mbio.03291-22)
Supplement: FIG S6 [file mbio.03291-22-s0006.pdf]

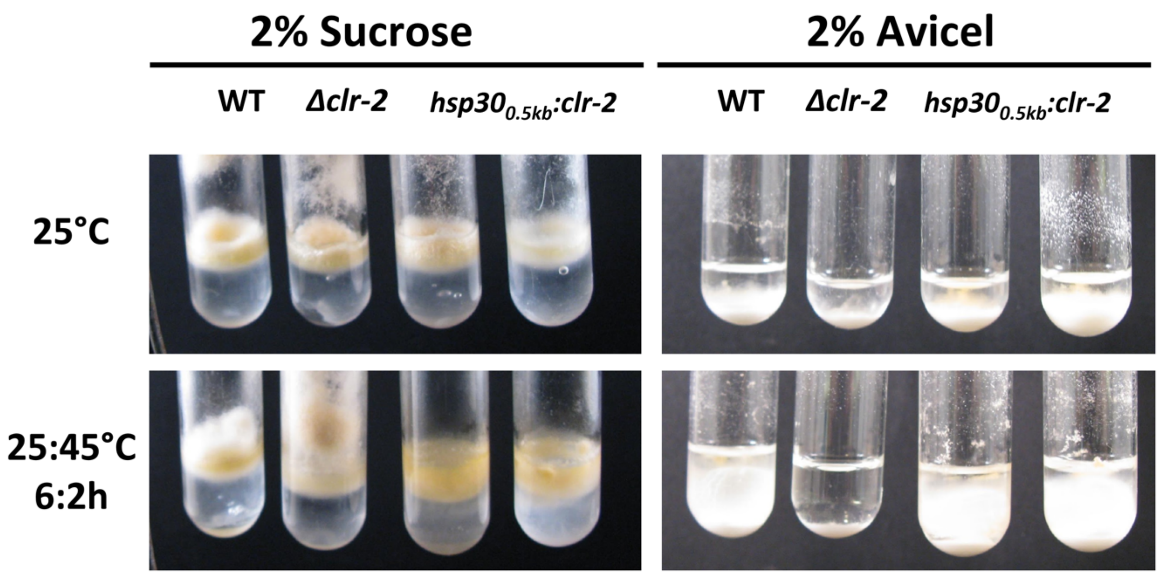

Figure S6. The *hsp30*$_{0.5kb}$ promoter can control a catabolic pathway of biotechnological interest. Conidia (10^6) from WT (x654-1), Δ*clr-2*, *hsp30*$_{0.5kb}$:*clr-2* (biological clones 1 and 2) were grown in Vogel's media with sucrose (2%w/v) and crystalline cellulose (Avicel, 2%w/v) as carbon source. One set of tubes grew at 25ºC, while the others were exposed to a cycle of 45ºC for 2 h and 25ºC for 6 h (repeated 3 times every 24 h). All tubes were placed in a shaker (125 rpm) in constant lights (LL), for 4 days (sucrose) or for 7 days (Avicel). The photographs are representative of the behavior of three independent experiments.
