# Supplementary material for: Developing a Temperature-Inducible Transcriptional Rheostat in Neurospora crassa
Source: mBio. 2023 Feb 6;14(1):e03291-22. doi: 10.1128/mbio.03291-22 (PMC9973361; doi:10.1128/mbio.03291-22)
Supplement: FIG S7 [file mbio.03291-22-s0007.pdf]

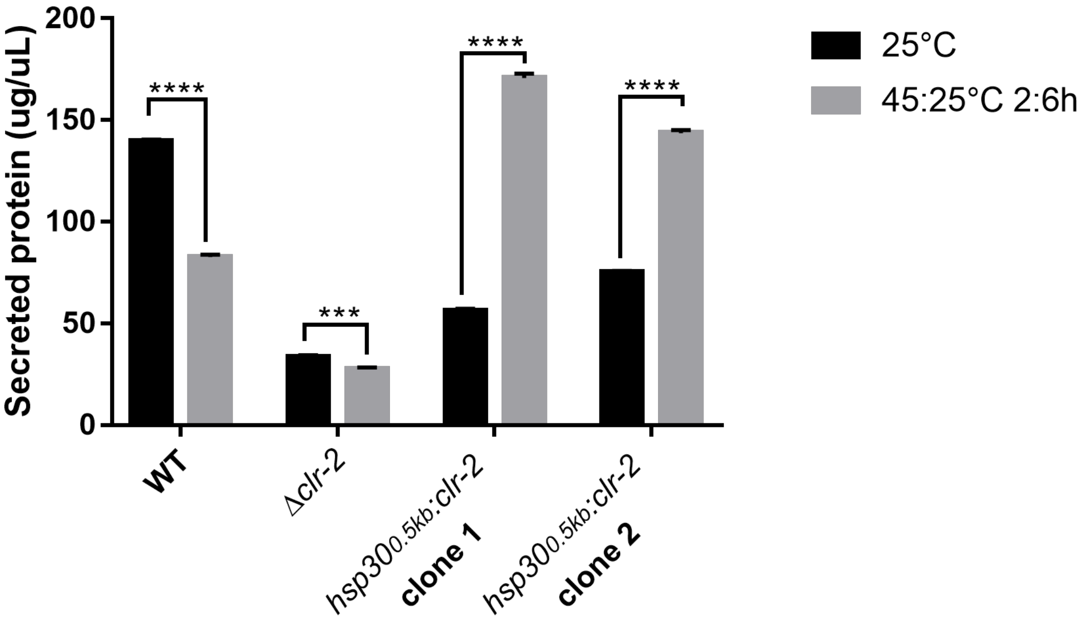

Figure S7. Secreted protein levels in shifted cultures. (**a**) Conidia (10^6) from WT (x654-1), *Δclr-2*, *hsp30$_{0.5kb}$:clr-2* (biological clones 1 and 2) were inoculated in Vogel's media with sucrose and were grown in constant light conditions (LL) at 25°C for 48h. Then mycelia were washed and transferred to Vogel's media with crystalline cellulose (Avicel, 2%w/v) as carbon source, and the flaks were grown in constant light conditions (LL) at 25°C with or without a high-temperature treatment (a pulse at 45°C for 2h every 6h; 25:45°C 6:2h). Supernatant protein concentrations were determined from 24 h cultures of WT, *Δclr-2*, and *hsp30$_{0.5kb}$:clr-2* strains grown on 2% Avicel with or without the heat-temperature treatment (25:45°C 6:2h) as explained in Material and Methods. The mean and standard deviation represent three independent measurements, in three independent experiments. Statistical significance was performed using a two-way ANOVA plus Sidak's test (*** = $p < 0.001$; **** = $p < 0.0001$).
