# Supplementary material for: Developing a Temperature-Inducible Transcriptional Rheostat in Neurospora crassa
Source: mBio. 2023 Feb 6;14(1):e03291-22. doi: 10.1128/mbio.03291-22 (PMC9973361; doi:10.1128/mbio.03291-22)
Supplement: FIG S8 [file mbio.03291-22-s0008.pdf]

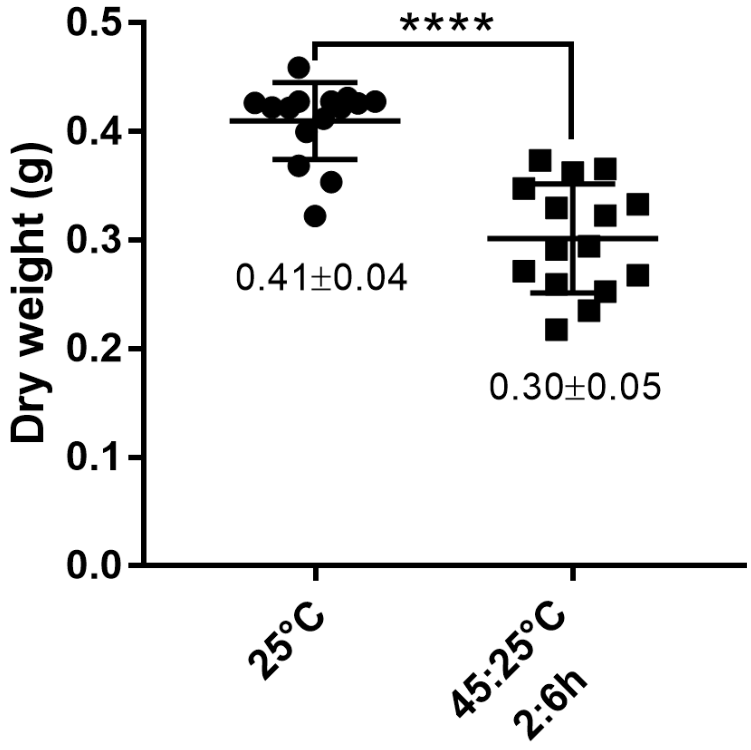

Figure S8. Effect of high temperature treatment in WT growth. Conidia (10^6) from WT (x654-1) was inoculated in Vogel's media with sucrose (2%w/v) as carbon source. Flasks were grown in constant lights (LL) at 25°C with or without a high-temperature treatment (a pulse at 45°C for 2h every 6h, 25:45°C 6:2h). Cultures were kept for 4 days in a shaker (125 rpm) and then the mycelium was harvested and dried. Statistical significance was determined by a t-student (**** = $p < 0.0001$).
