# Supplementary material for: Developing a Temperature-Inducible Transcriptional Rheostat in Neurospora crassa
Source: mBio. 2023 Feb 6;14(1):e03291-22. doi: 10.1128/mbio.03291-22 (PMC9973361; doi:10.1128/mbio.03291-22)
Supplement: FIG S9 [file mbio.03291-22-s0009.pdf]

>SP30A

aatacattgatcacctggaaccttcttgaatgctcttcagatcttatatcatctagtaaactagcagtaccaagctatcaaacctttacctcggcgtgaaat
taccagaaactgcgaccgggtgcagcgcagtgtggaactttctaggctgctcccgaagcgtgcgattggccagcgaattacagtggccattctagaatg
acctggcatctgcatcaaatgctccttccaccttcttcgtccttcgcatcacaggtgaaagacaaggtaatttgtgaagcaaacaatgctcccactcaaat
ataaatctgggtgtgatgtctcccttttcatattgtcgattctctgttcagcagatcaagatcatccagcaagcgaagtaatcactctgaacactctcaaca
gcatctactacactcagcaaacgcacagatacctccgtcgccactcttttaacacacctaaagtcaaaa

>SP30B

caaaacgggattcaatacattgatcacctggaaccttcttgaatgctcttcagatcttatacagctactacagtttcataatcagtctcatctagtaaacta
gcagtaccaagctatcaaaacaaactagttaggaaggaaatattccctttacctcggcgtgaaattaccagaaactgcgaccactgattgatccggca
aaactgaagcgggtgcagcgcagtgtggaactttctaggctgctcccgaagcgtgcgattggcctcacaccgtctccccaagagaactccagcgaatt
acagtggccattctagaatgacctggcatctgcatcaaatgctccttccaccttcttcgtccttcgcatcacaggtgaaagacaaggtaatttgtgaagca
aacaatgctcccactcaaatataaatctgggtgtgatgtctcccttttcatattgtcgattctctgttcagcagatcaagatcatccagcaagcgaagtaat
cactctgaacactctcaacagcatctactacactcagcaaacgcacagatacctccgtcgccactcttttaacacacctaaagtcaaaa

Figure S9. FASTA sequence of the *SP30A* and *SP30B* promoters. Sequence of both synthetic promoters (*SP30A* and *SP30B*). *hse* (red), the newly assigned *hse* (light-blue), spacers (black) and the putative minimal promoter of *hsp30* (deep red) are indicated.
